# Multiple renal angiomyolipomas with asymptomatic nontraumatic pulmonary fat embolus: A case report

**DOI:** 10.1002/ccr3.8616

**Published:** 2024-03-08

**Authors:** Hourieh Soleimani, Masoud Pezeshki Rad, Donya Farrokh, Ehsan Hassannejad, Asma Payandeh, Sepideh Zahedi, Neda Karimabadi

**Affiliations:** ^1^ Department of Radiology, Faculty of Medicine Mashhad University of Medical Sciences Mashhad Iran; ^2^ Department of Radiology, School of Medicine Birjand University of Medical Sciences Birjand Iran; ^3^ Faculty of Medicine Mashhad University of Medical Sciences Mashhad Iran

**Keywords:** angiomyolipoma, embolism, fat, pulmonary

## Abstract

Although retroperitoneal bleeding and massive hematuria are potential complications of angiomyolipoma (AML), the pulmonary embolism as a presenting symptom is extremely rare. It is important to be aware that benign AMLs can present with pulmonary fat embolism.

## 1 INTRODUCTION

The condition known as pulmonary fat embolism occurs, when fat globules are present in the pulmonary circulation. Bone fractures, orthopedic procedures, blunt trauma, liposuction, and fat grafting are among the common etiologies of pulmonary fat embolism.^1^, ^2^

Renal angiomyolipoma (AML) is a benign tumor typically consisting of fat, smooth muscle tissue, and vessels.^3^ Roughly 80% of AMLs are sporadic, with an average age of 43 at the time of presentation, and the majority of cases are detected between the ages of 40 and 60. Female predilection is apparent in sporadic cases, as evidenced by a female‐to‐male ratio of 4:1.^4^ The incidental detection of AML is common, and while mostly asymptomatic, a few cases may manifest symptoms such as pain or complications like spontaneous bleeding.^5^ Renal AML does not extend beyond the kidney. Nevertheless, there are rare instances, where AML may manifest as pulmonary fat embolism.^6^, ^7^ In this study, we present a case of a patient with an asymptomatic renal AML, which was fortuitously discovered alongside an incidental diagnosis of an asymptomatic fat embolism.

## 2 CASE PRESENTATION

### 2.1 Case history and examination

A 45‐year‐old man with diabetes and with no history of trauma and no history of surgery was admitted and examined due to mood disorders and depression, paraparesis of lower limbs, and urinary incontinence. Symptoms began 5 days before admission.

The physical examination revealed an increase in deep tendon reflexes (DTR) and impairment in proprioception. The muscle forces of lower limbs were 4/5 and the patient was found to have a GCS of 15. The patient exhibited stable vital signs, no complaints of dyspnea or chest pain, and no signs of tachypnea, tachycardia, or fever. There was no petechial rash in dermatological examination. Other neurological examinations also gave no abnormality.

### 2.2 Methods

**FIGURE 1:**
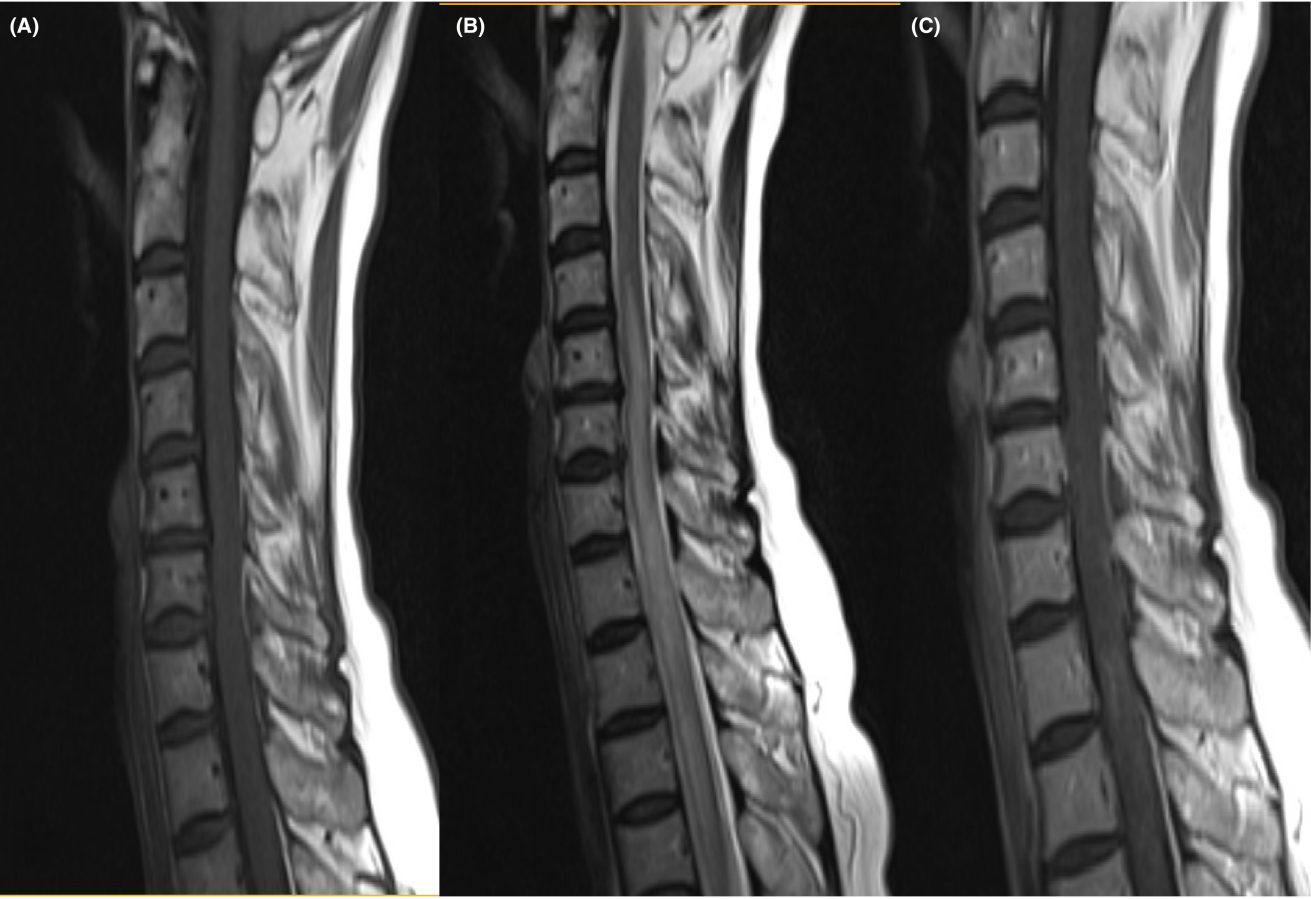
Cervicothoracic MRI scans show a long segment T1 iso signal (A) and T2 high signal (B) intensity between C4 and T5 level in the cervical cord with extension to the thoracic cord, with faint enhancement in some parts in the post contrast images (C).

MRI scans of the brain, cervical spine, and thoracic spine was performed. Brain MRI was normal. Cervicothoracic imaging revealed a long segment T1 iso signal and T2 high signal intensity between C4 and T5 level in the central part of the cervical cord with extension to the thoracic cord, demonstrating faint enhancement in some parts in the post contrast images (Figure 1).

**FIGURE 2:**
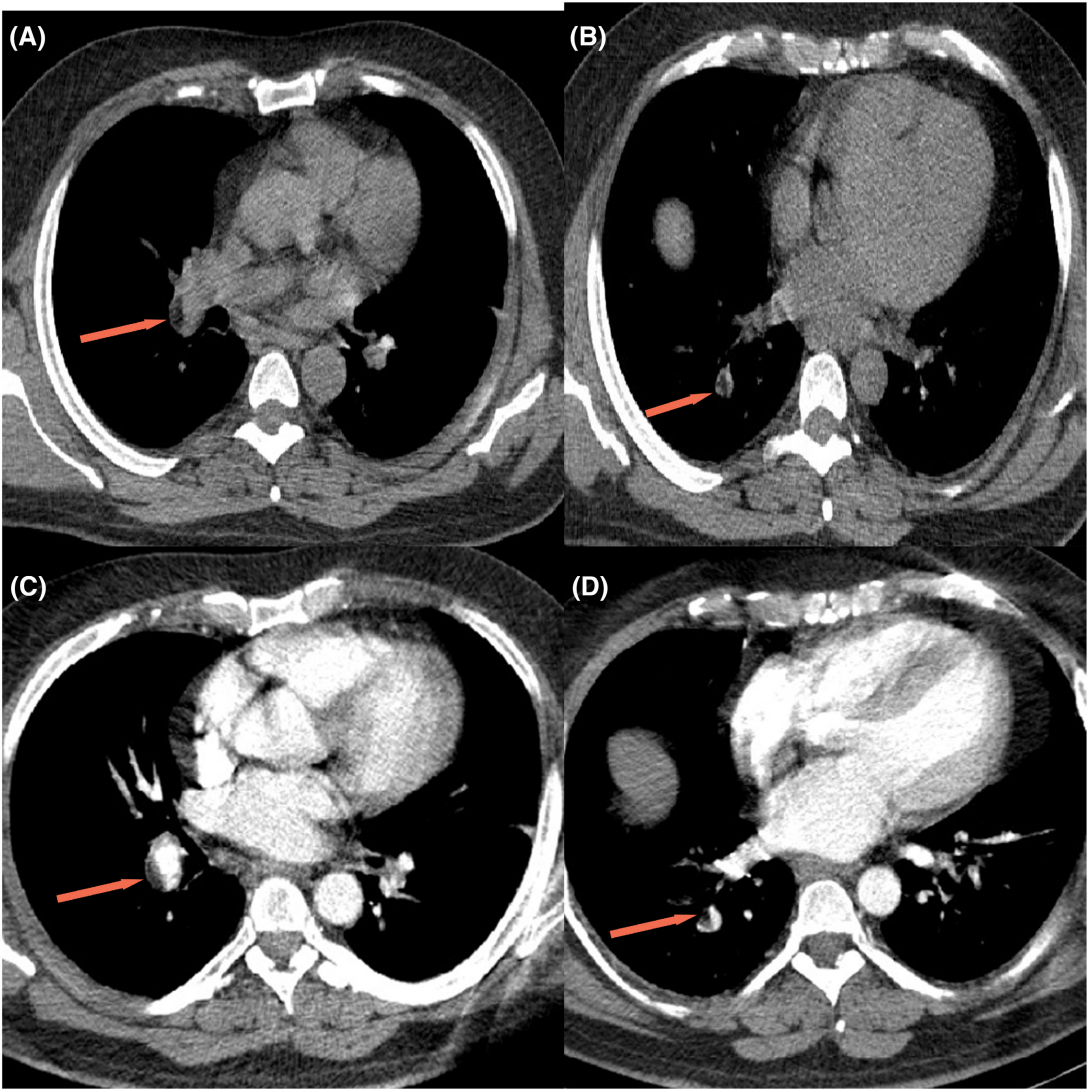
Non‐contrast chest CT scan (A, B) and pulmonary CT angiography (C, D) show intraluminal lesion with a fat density (arrows) in the right interlobar pulmonary artery, extending to the segmental branches of the right lower lobar, suggesting a fat embolism.

Following thorough neurological examinations and MRI scans, the patient was diagnosed with longitudinally extensive transverse myelitis (LETM). The evaluations for neuromyelitis optica, autoimmune encephalitis, and the ANA profile and tumor markers yielded negative results. In light of suspected paraneoplastic syndrome, the patient underwent a series of diagnostic tests, including a chest CT scan without contrast, an abdominopelvic ultrasound, and an abdominopelvic CT scan with IV contrast. The chest CT scan revealed an intraluminal lesion with a fat density located distally in the right interlobar pulmonary artery, extending to the segmental branches of the right lower lobar, suggesting a fat embolism. Pulmonary CT angiography was performed for further investigation, and the above findings were confirmed (Figure 2).

**FIGURE 3:**
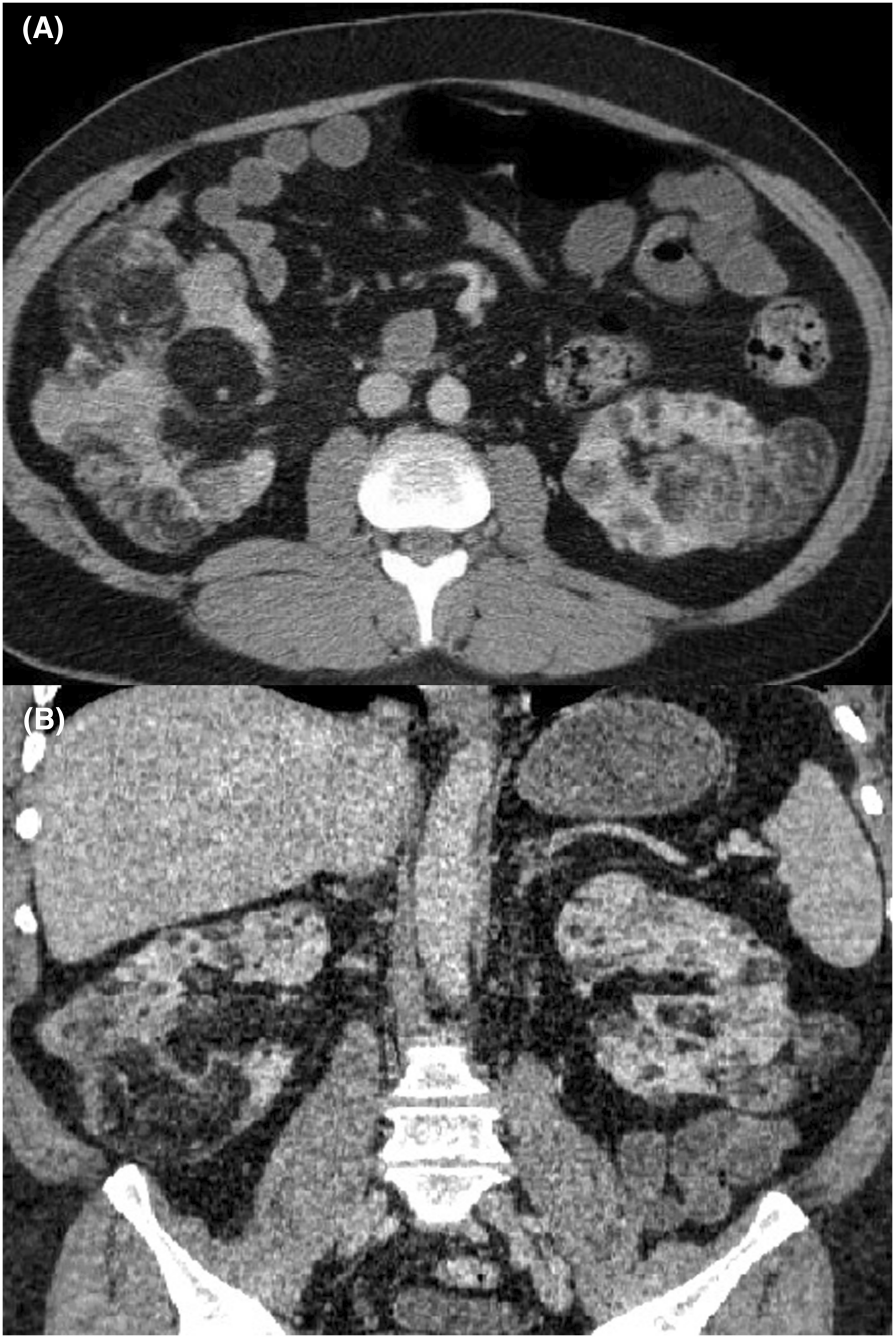
Contrast‐enhanced abdominal CT scan shows multiple renal masses in both kidneys, primarily consisting of adipose tissue in axial (A) and the coronal plane (B), without extension into the renal veins or IVC and with no enlarged lymph nodes.

The patient's abdominopelvic CT scan with IV contrast and ultrasound also revealed multiple renal masses in both kidneys, primarily consisting of adipose tissue (Figure 3). The masses did not extend into the renal veins or IVC. No enlarged lymph nodes were seen. A blood test performed showed normal serum BUN and Cr.

Based on these findings, it was determined that the patient had pulmonary fat embolus caused by AML.

Based on the LETM diagnosis, the patient underwent corticosteroid pulse therapy. The patient underwent the initiation of heparin treatment as recommended by the cardiology consultation.

### 2.3 Conclusion and results

After 5 days, the patient's hospital discharge was approved due to stable vital signs, good general condition, and a prescribed outpatient treatment regimen of Apixaban 5 mg BD for 3 months. He remained well at 6‐month post treatment follow‐up visit and physical examinations gave no abnormality.

## 3 DISCUSSION

AML refers to a benign mesenchymal tumor, which can occur as either a single or multiple masses. The occurrence of AMLs can be both sporadic and in association with tuberous sclerosis or lymphangioleiomyomatosis. The kidney is the primary site of involvement in the majority of cases.^6^ The majority of patients are asymptomatic and are typically detected incidentally on radiological imaging with characteristic findings. Biopsy is seldom necessary due to the typical imaging findings of AML.^8^ While retroperitoneal bleeding and massive hematuria are potential life‐threatening complications of AML, the occurrence of pulmonary embolism as a presenting symptom is extremely rare.^9^ Fat emboli are a known characteristic of a syndrome that is typically seen in cases of pelvic or long bone fractures, as well as orthopedic instrumentation.^1^

There have been only a few reported cases of renal AML associated with fat emboli. Symptomatic respiratory or cardiovascular manifestations were observed in the majority of cases, leading to treatment strategies that varied from observation to surgical embolectomy.^10^, ^11^, ^12^ Shinohara et al. experienced the most severe situation when an 83‐year‐old woman presented with acute hemodynamic shock and subsequently passed away within a few days.^13^ In certain cases, asymptomatic individuals were incidentally diagnosed.^14^

In the presented case, the absence of clinical evidence for fat embolism syndrome necessitated a diagnosis relying solely on the incidental identification of right lung fat density on a chest CT scan without contrast. In addition, in contrast to similar studies, our case did not present any evidence of AMLs extending to the renal vein or inferior vena cava. The patient remained free from respiratory distress symptoms throughout their hospitalization and was released on the fifth day.

In our specific case, the fat embolism was relatively minimal, potentially explaining the absence of symptoms in our patient upon presentation. It is possible that previously documented cases were of larger size or more centrally located, which triggered symptoms and necessitated additional investigation leading to the diagnosis of fat embolism. Our patient represents a rare case of male patients with AML accompanied by pulmonary fat emboli unrelated to tuberous sclerosis or lymphangioleiomyomatosis. As a result, it brings up the issue of including routine chest CT scans in the assessment for renal AML. Nonetheless, the long‐term consequences of diagnosing asymptomatic fat emboli remain uncertain unless symptoms are present.

This case showcased an uncommon example of a renal benign tumor (AML) that was accompanied by an asymptomatic pulmonary embolism. It is important to note that benign AMLs can exhibit such behavior. That being said, the long‐term consequences of diagnosing asymptomatic fat emboli remain unclear unless symptoms are present.

## AUTHOR CONTRIBUTIONS

**Hourieh Soleimani:** Conceptualization; data curation; project administration; supervision; writing – original draft; writing – review and editing. **Masoud Pezeshki Rad:** Data curation; writing – original draft. **Donya Farrokh:** Conceptualization; data curation. **ehsan hassannejad:** Data curation; writing – original draft; writing – review and editing. **Asma Payandeh:** Data curation; writing – original draft; writing – review and editing. **Sepideh Zahedi:** Data curation; writing – original draft. **Neda Karimabadi:** Conceptualization; data curation; visualization; writing – original draft; writing – review and editing.

## FUNDING INFORMATION

No fund was available for this study.

## CONFLICT OF INTEREST STATEMENT

The authors declare no conflict of interest in this study.

## ETHICS STATEMENT

The patient has provided written informed consent for the publication of this case report.

## CONSENT

Written informed consent was obtained from the patient to publish this report in accordance with the journal's patient consent policy.

## Data Availability

The data that support the findings of this study are available on reasonable request from the corresponding author. The data are not publicly available due to privacy or ethical restrictions.
